# Chinese first-year undergraduates’ strategy use in the English writing from sources task: influences from genders, critical thinking, and L2 proficiency

**DOI:** 10.3389/fpsyg.2023.1290312

**Published:** 2023-11-21

**Authors:** Wei Liu, Pengfei Zhao

**Affiliations:** ^1^College of Foreign Languages, Jilin Agriculture University, Changchun, Jilin, China; ^2^Department of Chinese Language Studies, The Education University of Hong Kong, Tai Po, Hong Kong SAR, China

**Keywords:** English writing from sources, writing strategy use, critical thinking ability, L2 proficiency, gender difference

## Abstract

English is widely used as a *lingua franca* in academic settings, including academic writing, in the modern age. When faced with complex writing tasks that involve multiple sources, the ability to effectively employ writing strategies becomes crucial for achieving writing success. This is particularly true for first-year university students who are learning English as a foreign language. Although previous studies have examined various individual difference factors that influence students’ use of source-based writing strategies, such as L2 proficiency and gender, there is a lack of research exploring the impact of critical thinking skills on students’ strategy use. To address this gap, the current study utilized a convenience sampling procedure to involve 526 first-year EFL undergraduates from six classes in mainland China. A writing task and questionnaire were employed to investigate the students’ critical thinking skills and strategy use during the English writing from sources task. Furthermore, the study examined whether there were differences in strategy use based on gender, L2 proficiency groups, and levels of critical thinking ability. A three-way MANOVA was conducted, revealing significant variations in the students’ writing strategy use based on gender, L2 proficiency groups, and critical thinking levels. Notably, interaction effects between critical thinking ability and gender were also observed. The study discusses important implications, emphasizing the need for teachers to integrate critical thinking and strategy training into practical writing classes, and to consider the diverse learning needs of different groups of students.

## 1 Introduction

Writing from sources in English has been recognized as a complex yet vital skill for university students, as it plays a crucial role in acquiring disciplinary knowledge in real-world academic learning (Cumming et al., 2016, 2018; Wette, 2020). This sort of skill is especially important for first-year undergraduates who are novices to the academic community, as they encounter multiple sources of information in their disciplinary courses (Von Der Mühlen et al., 2016; Wette, 2018). However, writing from sources also presents challenges for students’ literacy development, such as evaluating the credibility of source content and synthesizing information into a new text in a coherent manner (Plakans and Gebril, 2013; Wette, 2020). These challenges may be even more daunting for those who are learning English as a foreign language, given their limited language proficiency and lack of strategic skills (Plakans and Gebril, 2013; Zhu et al., 2021). In order to tackle this demanding writing task, L2 writers have been reported to employ certain strategies (Yang and Plakans, 2012; Yang, 2014; Shi et al., 2020). Nevertheless, previous studies have documented instances of inappropriate strategy usage among novice L2 writers, including directly copying information from sources or engaging in patchwriting (e.g., Shi, 2004; Keck, 2014).

Furthermore, due to the sheer number of English learners in China as well as the practical difficulties encountered among them during writing, Chinese students’ English writing from sources for academic purposes has risen as a critical issue in writing research, which has recently attracted remarkable attention from international researchers and educators (Cumming et al., 2018; Cheong et al., 2019; Zhu et al., 2021). Chinese L2 English students’ strategy use during source-based writing has remained a central topic over recent decades (e.g., Yang and Plakans, 2012; Yang, 2014; Cheong et al., 2019; Shi et al., 2020). In spite of previous endeavors, our understanding of the individual difference factors underlying students’ strategy use during English writing from sources remains insufficient so far. Therefore, it appears valuable to conduct more empirical research in this line of inquiry. To address this concern, the present study aimed to narrow the research gap by capturing a fuller picture of Chinese first-year L2 undergraduates’ strategy use during English writing from sources and delving into how exactly individual difference variables such as critical thinking, L2 proficiency, and gender may interact with each other in influencing students’ strategy use in the Chinese context.

## 2 Literature review

Language learning strategies have been a prominent and enduring focus in the field of L2 learning and teaching for several decades (Cohen and Wang, 2018). This fascination with strategies is largely predicated on the notion that using strategies may facilitate students’ language learning (Oxford, 1990; Macaro, 2006), which has garnered significant attention from researchers and educators across various language domains, including reading (e.g., Plakans, 2008) and writing (e.g., Golparvar and Khafi, 2021). Among the diverse range of literacy activities, the use of strategies by L2 writers during English source-based writing has increasingly aroused empirical attention thus far.

### 2.1 Strategy use in the writing from source tasks

Similar to traditional independent writing tasks that are based solely on writing prompts, writing from sources also involves the utilization of planning, monitoring, and reviewing strategies (Segev-Miller, 2007; Plakans, 2008, 2010; Mateos and Solé, 2009; Hayes, 2012). Planning, as defined by L1 and L2 writing researchers, encompasses the writer’s attention to idea generation, organization, and goal setting (e.g., Flower and Hayes, 1981; Kellogg, 1996; Johnson et al., 2012). Monitoring refers to the abstract mental process of regulating writing processes to ensure the maintenance of writing goals (Stein, 1990; Segev-Miller, 2007). In simpler terms, it involves writers comparing their developing texts to their intended writing goals (Johnson, 2020). Reviewing, referred to as evaluation by some L2 scholars (e.g., Yang and Plakans, 2012; Yang, 2014), involve writers reviewing and evaluating their proposed ideas and written texts (Hayes, 2012; Abdel Latif, 2021).

In addition to the previously mentioned important writing strategies, writing from sources involves distinct strategic categories, namely the synthesis and actual use of source information in new texts. This is because writers are required to draw from multiple source materials in order to compose a new text (Plakans, 2010; Knoch and Sitajalabhorn, 2013; Plakans and Gebril, 2013). Specifically, students need to transform the information from source materials into their own written texts through the processes of selecting (choosing specific semantic content from sources to include in their writing), connecting (establishing connections among the given materials to ensure flow, continuity, and interdependence between content), and organizing (disassembling and restructuring the source materials to generate a new organization; Spivey, 1997; Nelson and King, 2022). Additionally, the use of source materials in the written text has been recognized as an important strategy, particularly among L2 writers (e.g., Shi, 2004; Plakans and Gebril, 2013; Yang, 2014; Wette, 2018). For example, previous studies have shown that L2 writers seek language support by copying and paraphrasing linguistic expressions from source texts during writing, which facilitates their text generation (Shi, 2004; Keck, 2014; van Weijen et al., 2018). Despite the extensive literature on L2 learners’ use of strategies in writing from sources, there is still a lack of research in China that specifically addresses English academic writing based on sources for knowledge acquisition among university students, especially those in the introductory stage of higher education (Cumming et al., 2018).

Previous studies have extensively established the importance of writing strategies in students’ L2 source-based writing achievement (e.g., Yang and Plakans, 2012; Golparvar and Khafi, 2021). For instance, Yang and Plakans (2012) found that the usage of discourse synthesis by Chinese EFL undergraduates significantly predicted their performance in integrated writing tasks. Similarly, van Weijen et al. (2018) discovered that the source use behavior of Dutch L2 learners significantly predicted the quality of their written texts. Given the significance of strategy use in English writing, numerous research studies have explored potential influential factors that underlie students’ strategy use in L2 writing, such as language proficiency (e.g., Raimes, 1987; Chien, 2012), and critical thinking skills (Esmaeil Nejad et al., 2022; Teng and Yue, 2023), and gender (e.g., Bai et al., 2020; Alfarwan, 2021). However, the findings from these studies have varied, highlighting the complex nature of strategy use in L2 writing.

### 2.2 Contributing factors of strategy use in L2 writing

Over the past few decades, there has been a growing body of evidence on the influence of individual difference factors, such as gender, L2 proficiency, and critical thinking skills, on L2 writing strategy use. However, most of these studies have primarily focused on independent writing activities, leaving a dearth of research on the potential effects of these factors on students’ strategy use in source-based writing tasks. In the following review, we will attempt to explore the relevant studies that have examined these variables of interest individually.

#### 2.2.1 Gender

Previous research on the influence of gender on L2 writing strategies has yielded mixed findings. For example, Liyanage and Bartlett (2012) used Oxford’s (1990) Language Learning Strategy questionnaire and found significant gender differences in metacognitive and cognitive strategies among L2 secondary students in Sri Lanka. Female students were found to employ self-evaluation and planning strategies more frequently than their male peers, while no significant differences were found in self-monitoring strategies. In terms of cognitive strategies, male students demonstrated a higher frequency of rehearsal and translation strategies, but showed less preference for reduction and resourcing strategies compared to females.

Similarly, De Smedt et al. (2018) found significant gender differences in thinking, planning, revision, and control strategies for informational and narrative writing among Flemish fifth-and sixth-grade students. Female students exhibited a higher frequency of using these strategies compared to male students during writing. De Smedt et al.’s findings were partially supported by Bai et al.’ (2020) recent study that documented significant gender differences in the use of planning, acting on feedback, and self-initiation strategies during English narrative writing among primary students in Hong Kong. Female students relied more on these strategies compared to male students. However, no significant gender differences were found in the use of text-generating, self-monitoring, and revising strategies. More recently, Bailey and Almusharraf (2022) revealed that gender was significantly correlated with L2 Korean undergraduates’ monitoring and translating strategies, but they did not illustrate the differences in specific strategy use between females and males.

In contrast to previous evidence, Nia and Shahsavar (2019) found no significant differences in prewriting strategies (listing, mapping, and freewriting) during English academic writing between male and female L2 undergraduates in Iran. The inconsistency in previous findings may be attributed to different student populations and the specific writing tasks used in each study. It is important to note that the studies mentioned above focused on different aspects of writing and employed various measures and contexts. When it comes to more complex source-based English argumentative writing, it remains unclear whether female L2 university students use strategies differently from male students during the writing process. Further research is needed to explore this specific context and shed light on potential gender differences in strategy use among L2 university students in source-based writing tasks.

#### 2.2.2 L2 proficiency

There have been extensive empirical efforts to investigate the relationship between language proficiency and L2 learners’ strategy use, as outlined in a review by Tiryakioglu et al. (2019). For example, Bai et al. (2014) conducted a large-scale investigation of writing strategies used by Singapore primary students and found significant main effects of language proficiency on planning, text-generating, monitoring and evaluating, revising, and resourcing strategies. Advanced L2 learners were more likely to employ these writing strategies more frequently compared to their low-proficient counterparts. In a similar vein, Bai et al. (2020) recently indicated moderate associations between Hong Kong primary students’ English writing proficiency and planning, text-generating, self-monitoring, and revising strategies. They also found small associations for acting on feedback and self-initiation strategies. Specifically, low-proficient L2 writers used strategies less frequently during English narrative writing compared to medium-and high-level writers. However, the differences in writing strategy use between medium-and high-level groups were not significant.

While many studies have shown a relationship between language proficiency and L2 learners’ strategy use in writing, there are also researchers who have found that language proficiency does not necessarily affect problem-solving behaviors or mental processes in writing (Cumming, 1989). For example, some studies have found that language proficiency does not significantly influence self-instructions, goal setting, structuring, idea generation, metacomments, or formulation in L2 writing (e.g., van Weijen et al., 2009; Tillema, 2012). In a recent study by van Weijen et al. (2018) examining the relationship between source use, argumentation behavior, text quality, and L2 proficiency among Dutch L2 students, they found that L2 proficiency only had a significant influence on the relationship between argumentation behavior and text quality. Overall, the findings regarding the relationship between language proficiency and strategy use in L2 writing are still mixed. It is clear that more empirical research is needed to gain a comprehensive understanding of this relationship, particularly in the context of writing from sources in English. Different factors, such as the specific writing task and the complexity of the source-based writing, may also play a role in shaping the relationship between language proficiency and strategy use.

#### 2.2.3 Critical thinking skills

Critical thinking skills play a crucial role in L2 writing, particularly in tasks that involve writing from multiple sources (Mehta and Al-Mahrooqi, 2014; List and Alexander, 2019; Tarchi and Villalón, 2021; Tarchi et al., 2022). These skills can be seen as mental preparation for the execution of specific strategies during reading and writing activities (List and Alexander, 2019; Tarchi and Mason, 2020). However, there is limited empirical research specifically focusing on the relationship between critical thinking skills and writing strategy use in L2 writing. One notable study conducted by Teng and Yue (2023) investigated the relationships between critical thinking skills, metacognition (e.g., evaluating, monitoring), and Chinese university students’ English academic writing performance. They found that students’ metacognitive writing strategy use was significantly correlated with their critical thinking skills. This suggests that students with higher critical thinking abilities are more likely to employ effective metacognitive strategies in their writing.

To further explore the relationship between critical thinking abilities and writing strategy use, it may be beneficial to expand the literature review to the field of language learning in general. For example, Bagheri (2015) found significant positive correlations between critical thinking abilities and language learning among Chinese first-year EFL undergraduates. Nevertheless, Afshar and Movassagh (2017) investigated the relationships between critical thinking, language learning strategies and academic achievement among L2 university students in Iran. They revealed that students with different critical thinking scores did not perform differently on language learning strategies (i.e., memory, cognitive, metacognitive, social, affective, and compensatory strategies). This broader perspective can provide insights into the potential influence of critical thinking skills on strategy use in L2 writing tasks.

To sum up, more empirical studies are needed to fully understand the relationship between critical thinking abilities and writing strategy use in L2 writing, particularly in the context of writing from multiple sources. In writing from sources tasks, students are often required to analyze and evaluate the trustworthiness of documents for use in their writing (List and Alexander, 2019; Tarchi and Villalón, 2021; Nelson and King, 2022). This demands higher-order thinking skills, including critical thinking ability. Therefore, it is important to empirically investigate whether students’ critical thinking ability can affect their strategy use in L2 writing from sources tasks.

## 3 This study

According to the language education policy in China (Ministery of Education, China, 2022), there has been a growing emphasis on developing college students’ EFL writing ability, particularly in summarizing and integrating information from different sources, writing outlines, summaries, or abstracts, using complex sentences, and employing diverse articulation devices appropriately. As a result, source-based writing tasks have gained popularity in language assessment in the Chinese context (Shi et al., 2020). Examples include the continuation writing task in the English proficiency test for college entrance examination and argumentative writing from sources in the English proficiency test for English majors.

While existing studies have explored Chinese EFL students’ writing from source ability, focusing on areas such as strategy use and task construction, our understanding of the individual differences underlying Chinese L2 learners’ strategy use in writing from sources remains insufficient. Additionally, researchers have recently called for more attention from educators to Chinese students’ learning of English writing from sources. This is particularly crucial for first-year undergraduates who may have limited experience and knowledge of source-based writing, making the task of composing a coherent text in English based on multiple materials a challenging activity.

Therefore, this study aims to investigate the relationships between gender, language proficiency, critical thinking ability, and strategy use in the context of English writing from sources. The study seeks to provide significant implications for students’ development of source-based writing competence. To address our research concerns, three primary research questions have been proposed as follows:

1. What are the levels of Chinese L2 first-year undergraduates’ strategy use in English writing from sources task?
2. Are there significant differences in Chinese L2 first-year undergraduates’ strategy use in English writing from sources task in terms of students’ gender, L2 English language proficiency and critical thinking skills?
3. Is there any interaction effect among gender, L2 English language proficiency and critical thinking skills on Chinese L2 first-year undergraduates’ strategy use in the English writing from sources task?

## 4 Methods

### 4.1 Participants

In this study, we focused on the strategy use in English writing based on external source materials among first-year university students. A convenience sampling approach was adopted (Shirvan and Alamer, 2022), and a total of 535 first-year undergraduates (*Mean* = 18.28, *SD* = 0.78) from six classes at an average-level state-run university in northeast China participated in the study. The sample included 196 males and 339 females. All potential students received an invitation letter and were invited to participate voluntarily. The participants came from various disciplines, including biology, business, information, education, and others. After data screening, the sample was reduced to 526 cases, as 9 cases had random responses to all items (Pen et al., 2004). The students who took part in the study were compensated with credit for their English courses.

### 4.2 Instruments

#### 4.2.1 Questionnaire

The questionnaire used in this study consisted of three sections. The first section aimed to gather participants’ background information, including their name, gender, disciplinary background, and their English language proficiency scores in the College Entrance Examination. The second section was designed to measure students’ strategy use during source-based writing. It was adapted from Yang’s (2014) study, specifically tailored for L2 source-based writing. This section comprised three categories: evaluation (7 items), planning (3 items), and discourse synthesis (4 items). A five-point Likert scale ranging from “1″ (least used) to “5″ (very often used) was used to indicate participants’ frequency of strategy use. The internal consistencies of the four sub-scales of strategy use (i.e., discourse synthesis, planning, and evaluation) were 0.68, 0.87, and 0.63, respectively. The third section, adapted from Pen et al. (2004), focused on critical thinking. It included four constructs: truth-seeking (4 items), analyticity (3 items), systematicity (4 items), and critical thinking self-confidence (9 items). Participants responded using a six-point Likert scale, ranging from “1″ (strongly disagree) to “6″ (strongly agree). The internal consistencies of the four sub-scales of critical thinking (i.e., truth-seeking, analyticity, systematicity, and critical thinking confidence) were 0.64, 0.65, 0.70, and 0.82, respectively.

#### 4.2.2 English writing from sources task

In this study, a source-based argumentative writing task was developed based on previous literature (e.g., Plakans, 2008; Bråten and Strømsø, 2011; Yang, 2014). The task design followed the procedure outlined by Plakans (2008) for integrated writing tasks. The task itself consisted of a writing prompt and two English passages, which presented opposing stances on the topic of the application of AI in people’s everyday lives. The topic of the task was selected to be “*Application of artificial intelligence (AI) in people’s everyday life*,” as it is a debatable topic that is familiar to students in the modern age. The reading materials for the task were selected from various sources such as news articles or popular science texts, following the approach used by Bråten and Strømsø (2011). The first passage, titled “*Crime-fighting robot hits, rolls over child at Silicon Valley mall*,” was sourced from the Los Angeles Times and consisted of 504 words. The second passage, with a length of 383 words, discussed the application of AI in education. Both passages were chosen to have appropriate readability, as indicated by Flesch Kincaid reading ease scores of 46.5 and 58.4, respectively. To ensure the validity of the task, a panel meeting was organized, including the researchers and two experienced college English teachers. During this meeting, the task was refined, and it was collectively agreed upon that the difficulty level of the task was appropriate for the target participants.

### 4.3 Data collection

Due to the COVID-19 pandemic, the data collection procedure was adapted to an online format using the VooV meeting platform.^1^ The data collection process was conducted class by class in an online meeting environment. First, participants were asked to write an argumentative essay in English, with a maximum word limit of 200 words, within a time frame of 40 min. This session took place on a popular online writing website called Pigaiwang,^2^ which is commonly used in local universities for writing assessments. After completing the writing task, participants were then directed to respond to the questionnaire, which had been transformed into an online version using the Wenjuanxing platform.^3^ This ensured that the questionnaire could be easily accessed and completed by the participants in an online format. To ensure the quality of the data, both researchers and the teacher of each class served as examiners during the data collection process, which helped to maintain consistency and accuracy in the data collection procedure.

### 4.4 Data analysis

The data collected were transformed into SPSS 27.0 and Mplus 8.0 for data analysis. First, the internal consistency and confirmatory factor analysis (CFA) were performed to ensure the reliability and validity of the instruments. To indicate the good fitness of the model, the Chi-square statistic (*χ^2^*), Comparative Fit Index (CFI), and Root Mean Square Error of Approximation (RMSEA) were adopted in this study. If CFI were larger than 0.90 and RMSEA were smaller than 0.08, the model was considered acceptable (Kline, 2015). CFA results revealed that the strategy use scale had acceptable psychometrical properties (*χ^2^* = 248.548; *df* = 73; *p* < 0.001; CFI = 0.939; RMSEA [90% CI] = 0.068 [0.059–0.077]). Regarding the critical thinking scale, the fit indices showed that the model was an acceptable fit for the data: *χ^2^* = 405.532, *df* = 137, *p* < 0.001; CFI = 0.901; RMSEA [90% CI] = 0.064 [0.057–0.070].

Then the descriptive statistics were computed to indicate the characteristics of the variables of interest, including means, standard deviations, skewness, and kurtosis. According to Kline (2015), the obtained data presented a normal distribution with skewness values falling within the range of −2 to +2, and kurtosis values between −7 and +7. Subsequently, the three-way multivariate analysis of variance (MANOVA) was carried out to examine whether there existed significant differences in the strategy use for the integrated writing task, with participants’ gender, L2 proficiency and the mean of critical thinking abilities as independent variables (IVs) and the mean of writing strategy as dependent variables (DVs). In addition, the interaction effects of the three IVs were analyzed.

Prior to MANOVA, we classified students into two groups according to their reported English proficiency scores and three groups according to their critical thinking abilities using a two-step cluster analysis: 255 for the high-level group (*Mean* = 115.62) and 271 students for the low-level group (*Mean* = 91.16); 182 for the low-level group (*Mean* = 3.48), 254 for the medium-level group (*Mean* = 4.26), and 90 for the high-level group (*Mean* = 5.16). For the significant differences in strategy use across different levels of L2 proficiency and critical thinking ability, cross-comparisons were analyzed further by using the post-hoc test of Least Significance Differences (LSD) to determine where the differences exactly occurred. To show the strength of relationships between the IVs and DVs, partial eta squared (*η^2^*) was calculated.

## 5 Results

### 5.1 Chinese first-year undergraduates’ integrated writing strategy use

The descriptive statistics were presented in Tables 1, 2. As previous research defined (e.g., Oxford, 1990; Bai et al., 2020), the mean of strategy use in the range from 1.0 to 2.4 was considered to be low frequency, 2.5 to 3.4 medium frequency, and from 3.5 to 5.0 high frequency. Generally, the first-year undergraduates in this study showed a medium level of writing strategy use (*Mean* = 3.28, *SD* = 0.72). Among these strategies, students tended to make significantly more use of discourse synthesis with the highest frequency than other strategies (*Mean* = 3.38, *SD* = 0.80) according to the repeated measurement results [Wilks’ lambda = 0.791, *F*(3, 433) = 38.178, *p* < 0.05, *η^2^* = 0.209]. The second most frequent strategy was the evaluation (*Mean* = 3.35, *SD* = 0.86), which was significantly utilized more than planning and source use (*p* < 0.05). The planning was used least by students with the lowest frequency than other strategies (*Mean* = 3.02, *SD* = 0.93; *p* < 0.05).

**Table 1:** Descriptive statistics of primary variables.

	*Mean*	*SD*	Skewness	Kurtosis
DS	3.40	0.79	−0.095	−0.201
EVA	3.36	0.85	−0.230	−0.006
PLAN	3.02	0.93	0.037	−0.292
Strategy_Total	3.30	0.73	−0.078	0.065
CT_Total	4.14	0.63	0.405	−0.154
L2 Proficiency	103.02	15.13	−0.626	1.584

DS, Discourse synthesis; EVA, Evaluation; PLAN, Planning; CT, Critical thinking.

**Table 2:** Students’ writing strategy use across different genders, L2 proficiency levels and critical thinking groups.

Strategy	Gender				L2 proficiency				Critical thinking					
	Male (*N* = 190)		Female (*N* = 336)		High (*N* = 255)		Low (*N* = 271)		High (*N* = 90)		Medium (*N* = 254)		Low (*N* = 182)	
	*M*	*SD*	*M*	*SD*	*M*	*SD*	*M*	*SD*	*M*	*SD*	*M*	*SD*	*M*	*SD*
DS	3.29	0.82	3.45	0.76	3.52	0.80	3.27	0.76	3.94	0.91	3.49	0.66	2.99	0.69
EVA	3.11	0.81	3.51	0.83	3.51	0.83	3.22	0.84	3.01	0.81	3.56	0.84	2.97	0.75
PLAN	2.81	0.87	3.02	0.93	3.12	0.89	2.94	0.95	3.03	0.72	3.58	0.77	2.64	0.82
Overall	3.09	0.70	3.42	0.72	3.43	0.72	3.18	0.72	2.90	0.62	3.42	0.68	2.90	0.61

### 5.2 Differences in integrated writing strategy use in terms of gender, English proficiency, and critical thinking abilities

Following Huberty and Petoskey’s (2000) guideline, Box’s test was performed to check the hypothesis of homogeneity of covariance across groups, using *p* < 0.005 as a criterion. The results were insignificant, which indicated that the observed covariance matrices of the dependent variables were equal across groups (Box’s M = 113.732, *p* = 0.001). MANOVA can be performed accordingly and Wilks’ Lambda test was adopted. The results suggested that students used writing strategies significantly differently across genders [Wilks’ lambda = 0.938, *F*(4, 512) = 11.196, *p* < 0.05, *η*^2^ = 0.062], L2 proficiency [Wilks’ lambda = 0.986, *F*(3, 512) = 2.445, *p* < 0.05, *η*^2^ = 0.014], and critical thinking abilities [Wilks’ lambda = 0.794, *F*(6, 1,042) = 20.823, *p* < 0.05, *η*^2^ = 0.109].

As Dörnyei (2007) postulated, in terms of *η^2^*, three levels were generated to indicate the strength of effect sizes: small effect = 0.01, moderate effect = 0.06 and large effect = 0.14. By these criteria, a moderate association was observed between gender and writing strategy use, which was demonstrated by around 6.2% of the multivariate variance of writing strategy use related to gender variable (*η^2^* = 0.062). Approximately 1.4% of the multivariate variance of strategy use was responsible for L2 proficiency while a strong relationship between strategy use and critical thinking abilities with a large effect size (*η*^2^ = 0.109). Based on MANOVA results, the univariate ANOVA analysis was conducted on each strategic category. The assumption of homogeneity of variance for each strategy was testified using Levene’s test. No significant results were yielded with all *p*s > 0.05.

#### 5.2.1 Gender

Based on univariate ANOVA results, we found significant differences in discourse synthesis (*F* = 5.254, *p* < 0.05, *η*^2^ = 0.010), evaluation (*F* = 28.175, *p* < 0.05, *η*^2^ = 0.051), and planning (*F* = 17.241, *p* < 0.05, *η*^2^ = 0.032) between males and females (see Table 3). Despite the significant differences, the effect sizes were at a low level, which illustrated a small relationship between gender and writing strategy use. To be specific, gender was responsible for around 1%, 5.1%, and 3.2% of the multivariate variance of discourse synthesis, evaluation, and planning, respectively. It was indicated that female students tended to use the three writing strategies more frequently than their male peers.

**Table 3:** Between-subjects effects between genders.

Strategy	*df*	Mean square	F	*p*	Partial *η*^2^
DS	1	3.257	5.254	0.022	0.010
EVA	1	19.192	28.175	0.000	0.051
PLAN	1	14.344	17.241	0.000	0.032

#### 5.2.2 L2 proficiency

The ANOVA analysis results (see Table 4) showed that there existed significant differences in the writing strategies between high-and low-level L2 proficiency groups: discourse synthesis (*F* = 14.363, *p* < 0.05, *η*^2^ = 0.027), evaluation (*F* = 15.284, *p* < 0.05, *η*^2^ = 0.028), and planning (*F* = 5.392, *p* < 0.05, *η*^2^ = 0.010). By Dörnyei’s (2007) criteria, students’ L2 proficiency held a weak association with discourse synthesis, evaluation, and planning with partial *η*^2^ = 0.027, 0.028, and 0.010, respectively. To be specific, L2 proficiency accounted for around 2.7, 2.8 and 1% of the multivariate variance of discourse synthesis, evaluation, and planning, respectively. It was indicated that high-level L2 learners more frequently employed the three writing strategies than their low-level counterparts.

**Table 4:** Between-subjects effects across different L2 proficiency groups.

Strategy	*df*	Mean square	F	*p*	Partial *η*^2^
DS	1	8.754	14.363	0.000	0.027
EVA	1	10.660	15.284	0.000	0.028
PLAN	1	4.586	5.392	0.021	0.010

#### 5.2.3 Critical thinking abilities

As shown in Table 5, significant differences in three strategies were found among students at different levels of critical thinking ability: discourse synthesis (*F* = 55.951 *p* < 0.05, *η*^2^ = 0.176), evaluation (*F* = 41.829, *p* < 0.05, *η*^2^ = 0.138), and planning (*F* = 43.843, *p* < 0.05, *η*^2^ = 0.144). Based on partial *η*^2^, we found strong associations between discourse synthesis, evaluation and critical thinking abilities with *η*^2^ = 0.176 and 0.144, and nearly strong associations between evaluating and critical thinking ability with *η*^2^ = 0.138. Approximate 17.6% and 14.4% of the multivariate variance of discourse synthesis and planning were explained by students’ critical thinking ability, while 13.8% of the multivariate variance of evaluating was explained by critical thinking. The *post-hoc* test further indicated that students with high critical thinking abilities tended to use discourse synthesis, evaluation and planning more frequently than medium-and low-level critical thinking ability groups. In addition, the medium-level group used the three strategies more frequently than the low-level group (see Table 6).

**Table 5:** Between-subjects effects across different critical thinking ability groups.

Strategy	*df*	Mean square	F	*p*	Partial *η*^2^
DS	2	23.833	55.951	0.000	0.176
EVA	2	22.374	41.829	0.000	0.138
PLAN	2	27.131	43.843	0.000	0.144

**Table 6:** Post-hoc test for strategy use across different critical thinking ability groups.

Strategy	L2 proficiency level		Mean difference	*p*
DS	Low	Medium	−0.487	0.000
	Low	High	−0.946	0.000
	Medium	High	−0.459	0.000
EVA	Low	Medium	−0.503	0.000
	Low	High	−0.873	0.000
	Medium	High	−0.370	0.000
PLAN	Low	Medium	−0.447	0.000
	Low	High	−1.024	0.000
	Medium	High	−0.577	0.000

### 5.3 Interaction effect of gender and critical thinking abilities

**Figure 1:**
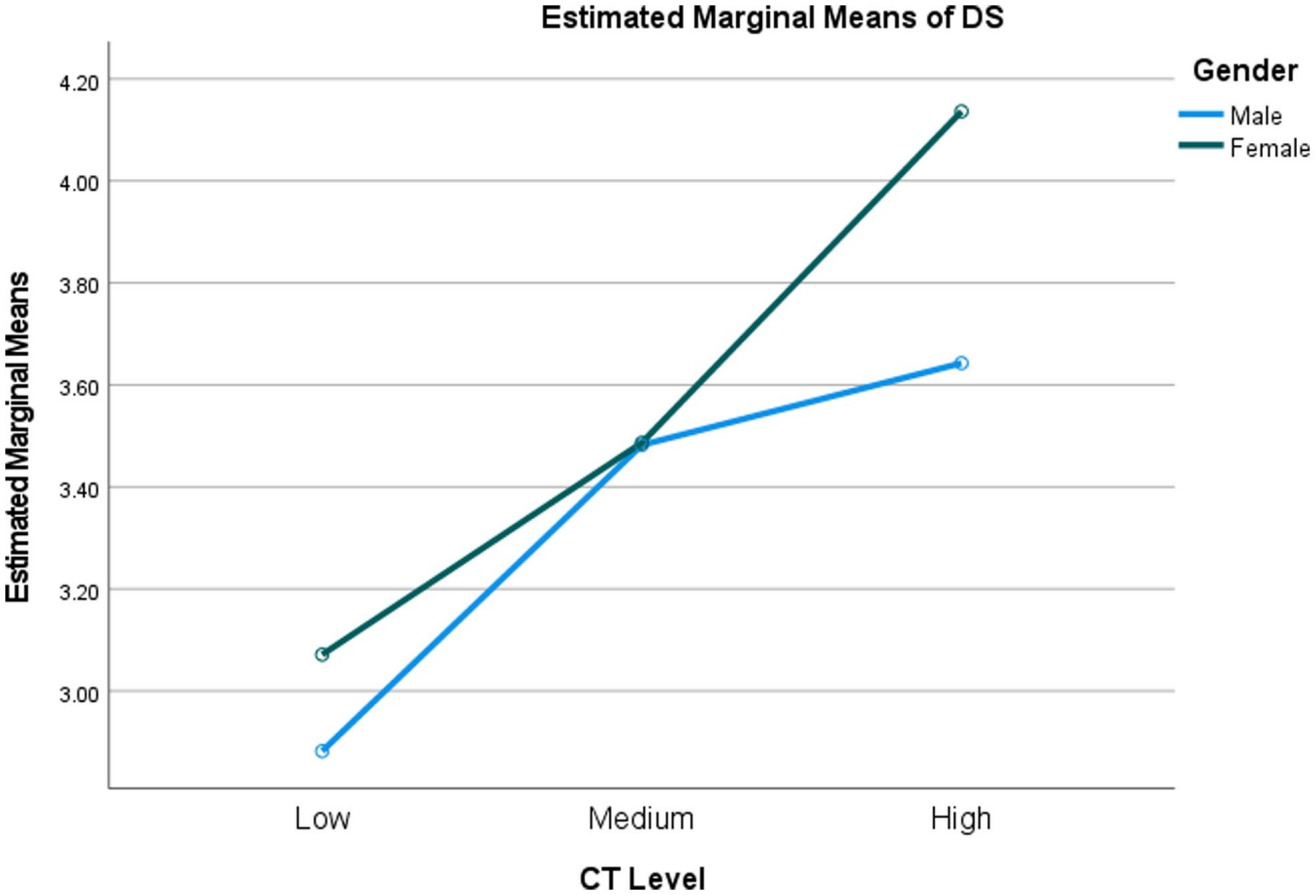
Interaction effect between gender and critical thinking on discourse synthesis. CT, critical thinking.

**Figure 2:**
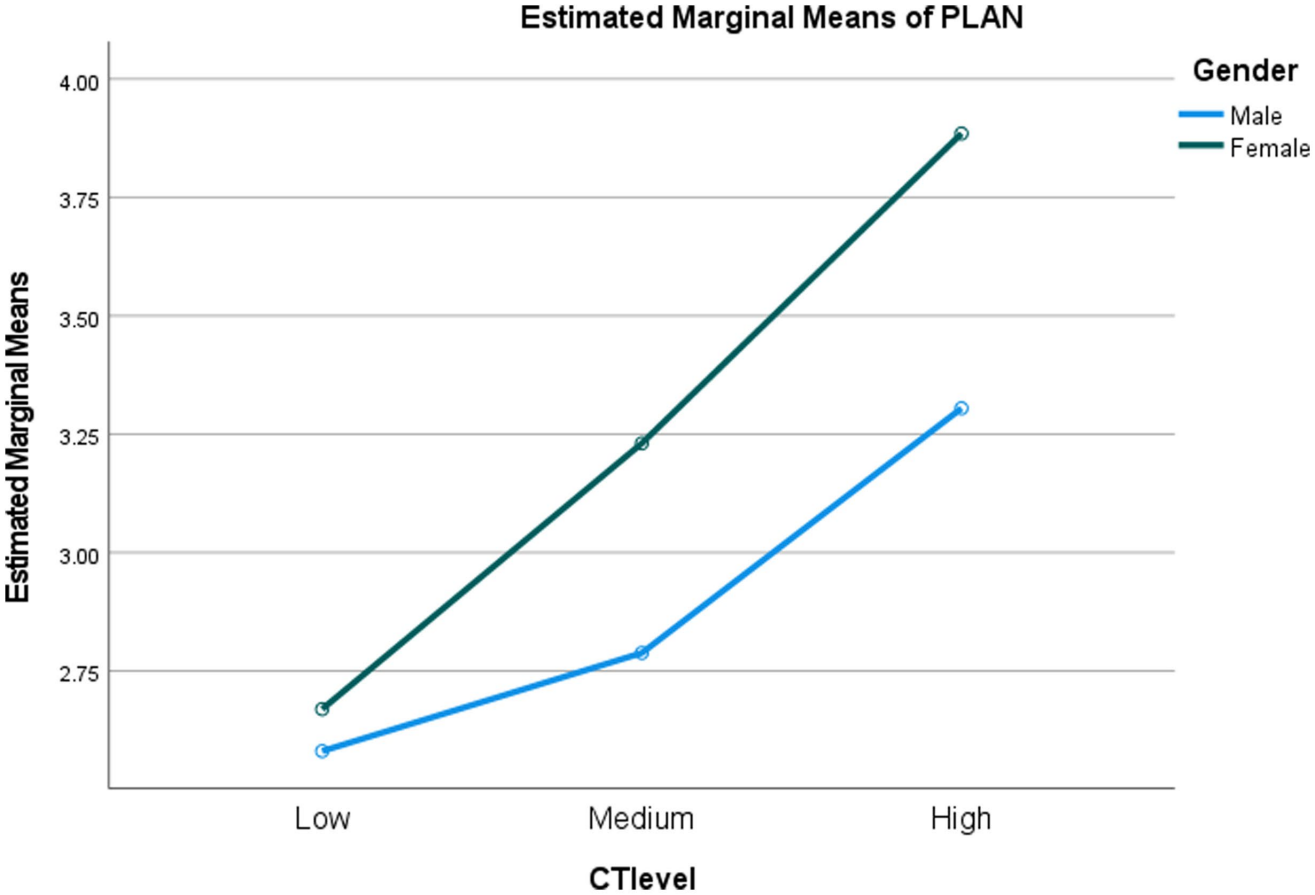
Interaction effect between gender and critical thinking abilities on planning.

Based on MANOVA results, we identified a significant interaction effect between gender and critical thinking abilities (see Table 7). It was found that a small interaction effect occurred in students’ usage of discourse synthesis (*F* = 2.697, *p* = 0.025, *η*^2^ = 0.014) and planning (*F* = 2.832, *p* = 0.041, *η*^2^ = 0.012; see Figures 1, 2), which illustrated that the effects of gender weakly intertwined with students’ critical thinking abilities. Around 1.4% and 1.2% of the multivariate variance of discourse synthesis and planning were explained jointly by critical thinking ability and gender. To be specific, as displayed in Figure 1, female students with high critical thinking abilities more frequently employed discourse synthesis than male students at the same level of critical thinking, whereas with students’ critical thinking ability increasing to medium level, female and male students showed similar frequency of discourse synthesis. Nonetheless, when it comes to the high level of critical thinking, female students used discourse synthesis more frequently than male students. Regarding planning strategy, the disparity between the two genders became increasingly large with the increase in students’ critical thinking abilities.

**Table 7:** Between-subjects effects for interaction between gender and critical thinking abilities.

Strategy	*df*	Mean square	F	*p*	Partial *η*^2^
DS	2	1.893	3.734	0.025	0.014
EVA	2	0.538	0.645	0.971	0.004
PLAN	2	2.274	3.222	0.041	0.012

## 6 Discussion

The aim of this study was to examine the strategy use of Chinese university students in English writing from sources and explore the potential influence of gender, language proficiency, and critical thinking on their writing strategy use. The study sought to contribute to a comprehensive understanding of how Chinese L2 learners utilize strategies in source-based writing and provide valuable insights for the teaching and learning of writing from sources.

### 6.1 Strategy use in English writing from sources among Chinese university students

In general, the first-year undergraduates in this study reported a medium level of writing strategy use. Such a tendency was also corroborated in previous literature concluding that Chinese students did not have frequent use of strategies in English writing (e.g., Yang and Plakans, 2012; Sun and Wang, 2020; Ye et al., 2021). It is interpretable that composing a well-organized text from complex source materials seems challenging for the majority of Chinese students due to the limited prior instruction and writing experience (Cumming et al., 2018). Specifically, discourse synthesis was used most frequently and planning was used least frequently among these strategies, which aligned with Ye et al.’s (2021) finding that Chinese L2 students mostly frequently used connection ideas from given sources during the English source-based narrative writing task.

However, it is interesting to note that Teng et al. (2022) found that Chinese EFL university students tended to use planning strategies more frequently than management of information strategies when faced with a graph-based English writing task. This discrepancy in findings may be attributed to the different task types used in these studies. Previous research has suggested that task types can influence students’ writing processes and strategy use (Plakans, 2008; Shi et al., 2020). In the present study, the task required students to compose an argumentative text based on reading materials. This type of task may elicit a higher frequency of discourse synthesis strategies, such as identifying or selecting useful information, connecting ideas across materials, and seeking language support (Shi, 2004; Plakans and Gebril, 2013; Keck, 2014). These strategies are more focused on integrating and synthesizing information from multiple sources, which may explain the difference in strategy use compared to the graph-based task in Teng et al.’s study.

On the other hand, the relatively low frequency of planning and evaluating revealed that students were strategically unskilled at controlling and regulating their thoughts or actions during the writing process (Zimmerman and Risemberg, 1997), for example, making writing outlines and generating ideas during writing. Although these metacognitive skills are crucial during writing (Hayes, 2012; Teng et al., 2022), they are seemingly cognitively demanding for L2 writers (Johnson, 2020). In addition, students’ strategy use during source-based writing may be related to their motivation such as writing self-efficacy (e.g., Golparvar and Khafi, 2021).

### 6.2 Gender

The present study revealed a gender disparity in the use of writing strategies among Chinese undergraduates in English writing from sources. Female students were found to significantly use discourse synthesis, evaluation, and planning strategies more frequently than male students. While there is limited literature on the gender effect on EFL students’ writing strategy use in source-based writing, our findings are supported by relevant studies on independent writing activities and general language learning (Green and Oxford, 1995; Liyanage and Bartlett, 2012; Bai et al., 2020).

Liyanage and Bartlett (2012) found that female high school students in Sri Lanka used planning and evaluation strategies more frequently than male students during English writing. Bai et al. (2020) also reported that Chinese primary students employed planning strategies more frequently than their male counterparts. One possible explanation for the gender difference in strategy use is that female students tend to be more motivated in English writing than male students (You et al., 2016). This higher motivation may contribute to their sophisticated cognitive processing and increased attention to the writing task (Bai et al., 2020; Cheong et al., 2022). Additionally, the social context may also play a role in the gender difference. As some researchers claimed, in the Chinese educational context, female students are often expected to excel in English learning compared to male students (Gu, 2002).

### 6.3 L2 proficiency

The findings of our study indicate a significant relationship between language proficiency and strategy use in the English writing from sources task. High-proficiency EFL learners in our study employed the three writing strategies more frequently compared to their low-proficiency peers. This finding is consistent with previous research (Raimes, 1987; Chien, 2012; Bai et al., 2014; Yang, 2014). High-proficiency students had a better command of L2 knowledge related to writing, allowing them to allocate more attentional resources to comprehending the meaning of source contents, synthesizing multiple pieces of information, monitoring the writing process, and reviewing their written texts. In contrast, low-proficiency writers often struggle with language expressions, such as lexical choice, due to insufficient vocabulary and grammatical knowledge in the L2 (Zhu et al., 2021). Consequently, low-proficiency writers may resort to negative writing strategies, such as translating from their L1 to the L2 (Baker and Boonkit, 2004).

Furthermore, as demonstrated in Chien’s (2012) study, proficient writers may possess a higher level of strategic awareness compared to low-proficiency writers. They are more likely to engage in recursive text revision, while less proficient writers may not recognize the importance of revising even if they are capable of doing it. Additionally, previous literature suggests that proficient L2 writers often begin writing without explicit plans, as they view planning as a non-unitary cognitive process, allowing them to temporarily set aside their thoughts and return to them if necessary (Bai et al., 2014; Bailey and Almusharraf, 2022).

### 6.4 Critical thinking ability

Critical thinking skills are crucial for students’ literacy development, although there is limited research available on this topic (Afshar and Movassagh, 2017; Cheong et al., 2022; Tarchi et al., 2022). It has been found that students with higher levels of critical thinking ability tend to use discourse synthesis, planning, and evaluation strategies more frequently, with a clear trend of high-level thinkers using these strategies more than medium-level thinkers, who in turn use them more than low-level thinkers. This association between critical thinking ability and writing strategy use is also supported by Esmaeil Nejad et al.’s (2022) research, which found a positive relationship between language learning strategies in English and critical thinking skills.

We interpret these findings to suggest that students with high critical thinking skills are able to critically comprehend and integrate information from source texts that express controversial viewpoints (Tarchi et al., 2022). This process requires them to compare and distinguish the different stances presented in the source materials, ultimately contributing to their own meaning construction (Tarchi and Villalón, 2021). Furthermore, critical thinking is often considered a set of higher-order thinking skills, including analysis, open-mindedness, and truth-seeking (Facione, 2011). Students with high-level critical thinking skills may therefore be more likely to employ demanding writing strategies that involve extensive cognitive processing, such as evaluation and planning. When students are dealing with multiple documents presenting different perspectives, those with high critical thinking abilities may evaluate the plausibility of the information in the sources based on their prior knowledge and then decide whether to integrate it into their written text based on its relevance to the topic (Von Der Mühlen et al., 2016).

### 6.5 Interaction effect of gender and critical thinking ability on writing strategy use

In our study, we found an interaction effect between critical thinking ability and gender on EFL university students’ writing strategy use, specifically in the areas of discourse synthesis and planning. Given the limited existing literature on this topic, we attempted to provide an explanation for this interaction effect based on findings from general academic learning research. Previous studies have indicated that female students generally have a higher level of critical thinking ability compared to male students (Salahshoor and Rafiee, 2016; Liu et al., 2019). As a result, female students may be more inclined to utilize their metacognitive skills, including planning, since critical thinking ability has been found to be positively correlated with students’ metacognition during the L2 learning process (Halpern, 1998; Ku and Ho, 2010). Critical thinking requires students to strategically utilize cognitive skills that are best suited for a particular situation, as well as actively control their own thinking processes to achieve task objectives (Ku and Ho, 2010). In our study, female students with high-level critical thinking ability may engage more cognitively in the English writing from sources task through more frequent usage of discourse synthesis and planning strategies to accomplish their writing goals.

## 7 Conclusion

The findings of the present study indicated that Chinese EFL undergraduates generally demonstrated a moderate level of writing strategies, with a particular emphasis on the frequent usage of discourse synthesis. Additionally, the study revealed that writing strategies were employed differently based on gender, L2 proficiency, and critical thinking ability. Notably, the interaction effect of gender and critical thinking ability on students’ writing strategy use was observed, with female students who possess high critical thinking ability exhibiting a higher frequency of discourse synthesis and planning strategies during writing compared to their male counterparts.

These findings have important pedagogical implications for the teaching and learning of source-based writing strategies among Chinese EFL university students. Firstly, it is crucial to enhance students’ awareness of the strategies necessary for source-based writing by incorporating strategy instruction into English writing classes (Zhu et al., 2021). Teachers should prioritize the training of sophisticated cognitive skills, such as evaluation and planning, as well as effective integration of information from multiple sources. Secondly, the individual differences in writing strategy use highlight the need for tailored strategy instruction that takes into account students’ developmental levels and learner characteristics (Bai et al., 2020). For instance, considering the strategic incompetency observed among male students with low critical thinking ability, more attention should be given to fostering students’ critical thinking skills rather than solely focusing on lexical and grammatical knowledge. Teachers can refine course design by incorporating training activities that promote critical thinking, such as comparing different ideas from documents or evaluating the credibility of source content.

It is important to note, however, that there are limitations in this study that should be acknowledged. Firstly, the participants were selected from a single university, which may limit the generalizability of the findings. Future research should aim to include a more diverse sample from different universities to enhance the external validity of the study. Secondly, while the use of questionnaires provides insights into general strategic patterns and influential factors, it is recommended to combine quantitative methods with qualitative approaches, such as interviews, to gain a deeper understanding of students’ perspectives and experiences with writing strategies. This would contribute to a more comprehensive understanding of the associations between gender, L2 proficiency, critical thinking ability, and writing strategy use among EFL university students. Finally, the cross-sectional design of the study limits the ability to establish causal relationships. Future research could adopt a longitudinal design to investigate the causal effects of critical thinking, L2 proficiency, and gender on strategy use in source-based writing over an extended period. This would provide valuable insights into the developmental trajectories of writing strategies and their relationships with critical thinking and gender.

## Data availability statement

The raw data supporting the conclusions of this article will be made available by the authors, without undue reservation.

## Ethics statement

The studies involving humans were approved by College of Foreign Languages, Jilin Agriculture University. The studies were conducted in accordance with the local legislation and institutional requirements. Written informed consent for participation in this study was provided by the participants’ legal guardians/next of kin. Written informed consent was obtained from the individual(s) for the publication of any potentially identifiable images or data included in this article.

## Author contributions

WL: Conceptualization, Data curation, Investigation, Project administration, Resources, Writing – original draft. PZ: Conceptualization, Formal analysis, Methodology, Software, Writing – review & editing.

## Footnotes

^1^ https://voovmeeting.com/

^2^ https://www.pigai.org/

^3^ https://www.wjx.cn/
